# Supplementary material for: Serial millisecond crystallography for routine room-temperature structure determination at synchrotrons
Source: Nat Commun. 2017 Sep 14;8:542. doi: 10.1038/s41467-017-00630-4 (PMC5599499; doi:10.1038/s41467-017-00630-4)
Supplement: Supplementary file 1 — Supplementary Information [file 41467_2017_630_MOESM1_ESM.pdf]

Description of Supplementary Files

File name: Supplementary Information
Description: Supplementary figures and supplementary tables.

File name: Supplementary Data 1
Description: Based on input parameters such as detector frame rate (Hz), nozzle size (µm) and jet flow speed (µm / s) The 'SMX calculator' spreadsheet allows to calculate experimental parameters like the distance each crystal travels during the exposure of a single frame, sample consumption and overall crystal exposure times.

File name: Peer review file

Supplementary Table 1. Comparing different beam sizes with equal overall flux.

Dataset	Lysozyme 5x5	Lysozyme 10x5	Lysozyme 20x5
Flux (ph / s)	3.9×10^{11}	3.6×10^{11}	4.2×10^{11}
Recorded patterns / Hits	60000 / 59772	60000 / 58783	60000 / 59539
Indexed patterns / Indexed patterns used	35471 / 27000	34662 / 27000	27071 / 27000
Resolution (Å)	24.84 – 1.58	24.84 – 1.58	24.84 – 1.58
Resolution CC < 0.5 (Å)*	1.50	1.58	1.59
Number of Reflections	8806396	9012259	9114606
Number of unique Reflections	18191	18186	18119
Redundancy	484.1 (17.0)	495.6 (15.6)	503.0 (11.8)
Completeness	99.8 (97.25)	99.7 (97.08)	99.3 (93.37)
I / sigma	8.35 (1.0)	7.77 (1.18)	6.93 (0.92)
*CC**	0.999 (0.72)	0.998 (0.55)	0.999 (0.19*)
CC1/2	0.996 (0.35)	0.993 (0.18)	0.995 (0.02)
Rsplit / Rmeas	5.43 (119.06)	5.91 (108.18)	6.64 (148.62)

* the sharp drop in CC* is due to the lack of data in the shell, resulting in the use of the "pushres" option in partialator, that applies a per-pattern resolution cutoff based on observed peaks

Supplementary Table 2. Comparing high frame rate and low frame rate data collection.

Dataset	$A_{2A}R$ 10 Hz	$A_{2A}R$ 25 Hz	$A_{2A}R$ 50 Hz
Indexed patterns	52074	101525	165709
Resolution	34-2.7	34-2.7	34-2.7
Number of Reflections	20692666	30363267	36043329
Number of unique Reflections	29159	29159	29159
Redundancy	709.6 (268.5)	1041.3 (321.8)	1236.1 (278.8)
Completeness	100 (100)	100 (100)	100 (100)
I / sigma	10.76 (0.87)	15.99 (1.79)	20.50 (3.07)
*CC**	0.99 (0.22)	0.99 (0.34)	0.99 (0.78)
CC1/2	0.98 (0.02)	0.98 (0.07)	0.99 (0.44)
Rsplit / Rmeas	11.27 (198.41)	11.09 (6846.27)	2.78 (36.51)

Supplementary Table 3. Minimal dataset for native SAD phasing.

Dataset	$A_{2A}R$ 5h 6keV
Collected images	1000000
crystals used	-
Indexed patterns	243537
patterns indexed (%)	24.4
Resolution	38.1 – 2.67
Number of Reflections	38979982
Number of unique Reflections	30220
Redundancy	1289.9 (120.5)
Completeness	100 (100)
I / sigma	23.19 (2.21)
*CC**	0.99 (0.18)
CC1/2	0.99 (0.02)
Rsplit / Rmeas	2.29 (93.02)

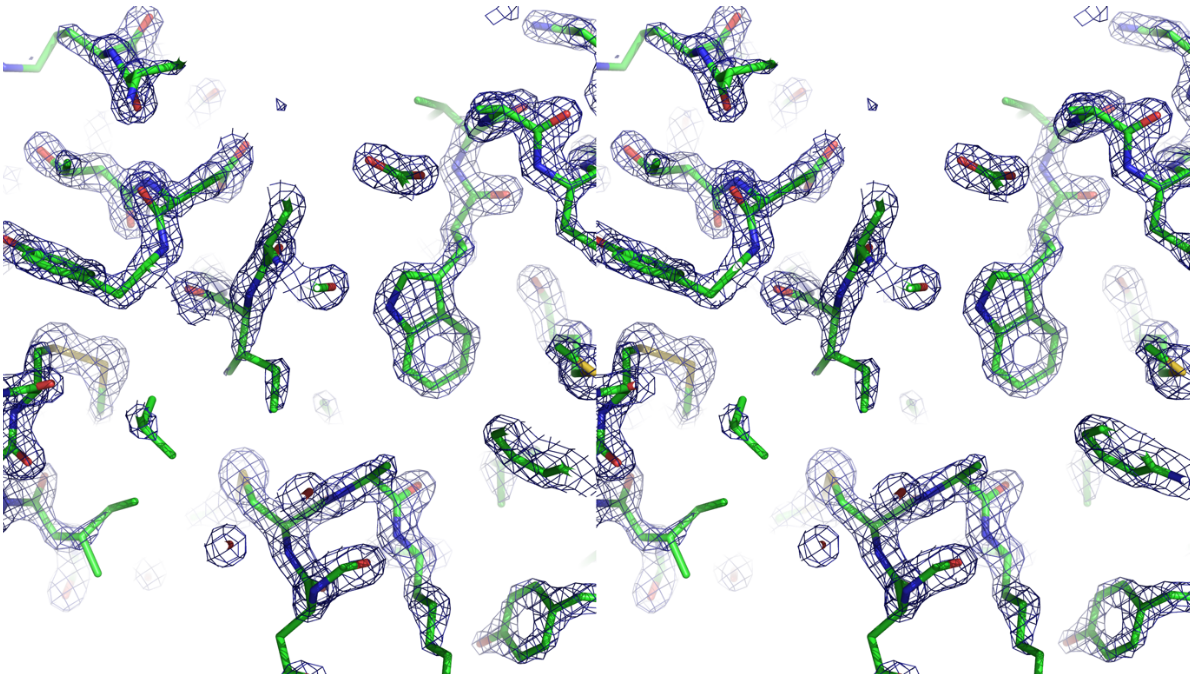

Supplementary Figure 1. Stereo View of lysozyme electron density including data to 1.5 Å. The electron density ($2F_oF_c$, 2.2σ) is of excellent quality and shows no signs of radiation damage.

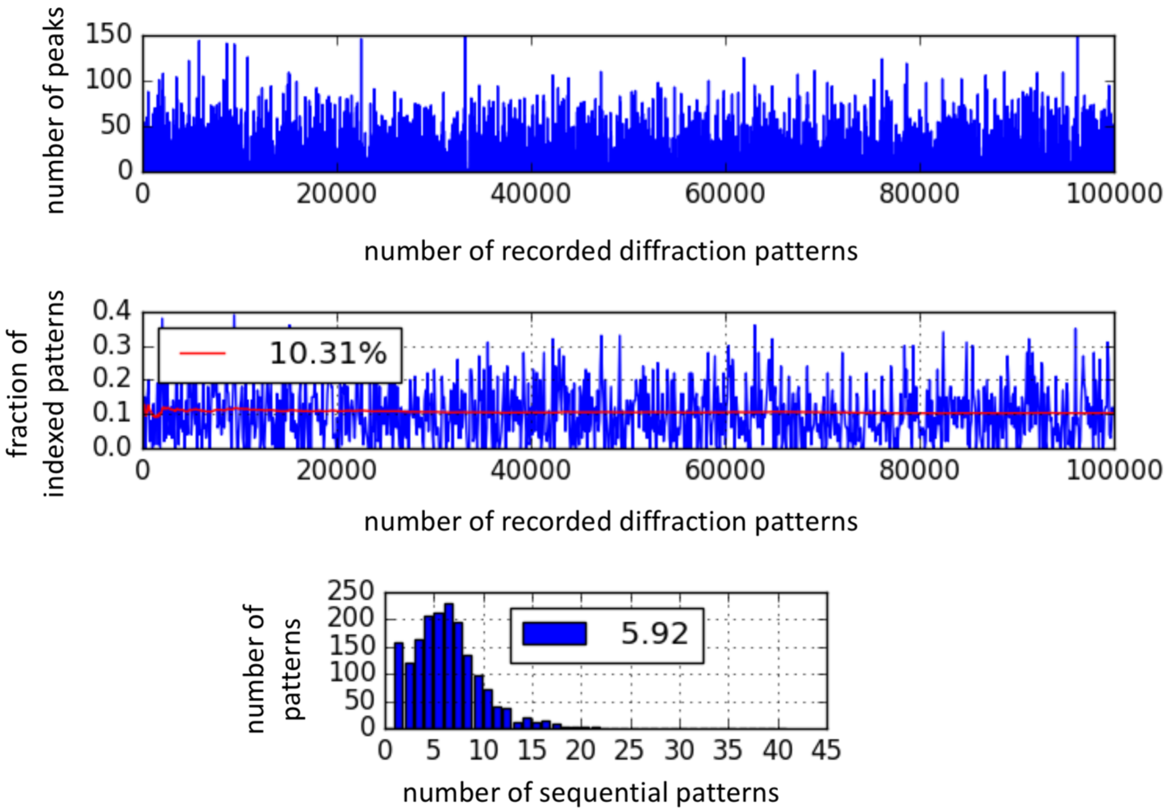

Supplementary Figure 2. Hitfinder live feedback. This representative run from our 6 keV data collection on the A_{2A} receptor shows from top to bottom: (I) the number of identified peaks per collected pattern; (II) the actual hit rate including only hits with at least 10 peaks with an I/σ of 10 (rolling average in blue and overall average in red); (III) number of sequential frames classified as hits before a non-hit is counted (this illustrates the crystal width; here each frame corresponds to a translation of 5 µm with the average crystal width of 5.92 frames and hence 30 µm corresponding to the average size of 20 – 40 µm of the A_{2A} receptor crystals used for the experiment).

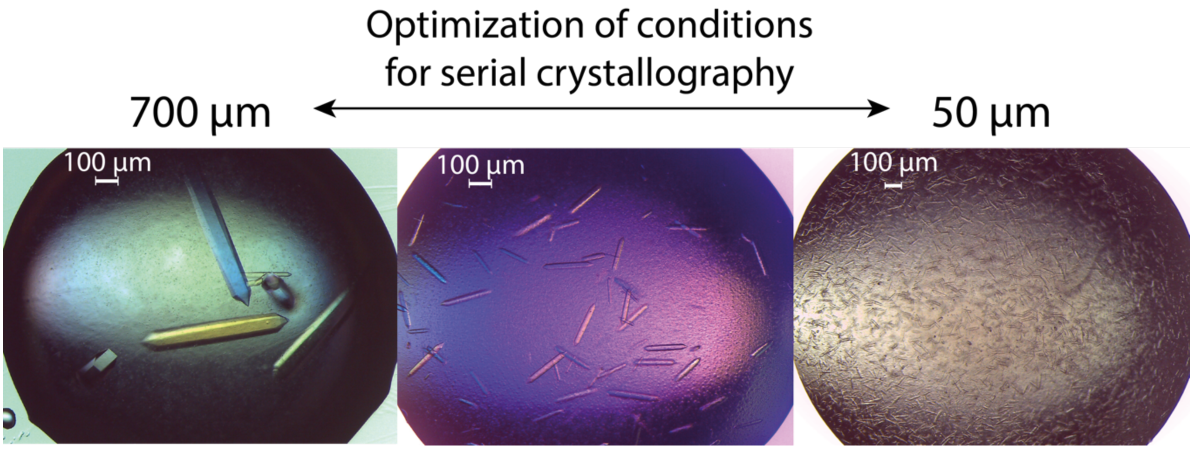

Supplementary Figure 3. MOSTO crystal optimization. Optimization of MOSTO crystals was straightforward. The protein and precipitant concentrations were increased until a shower of small crystals, most of them no larger than 50 µm were generated. There was only one round of optimization necessary, consuming 2.4 mg protein that were required to set up 2 x 24 well plates with 2 + 2 µl drop size.

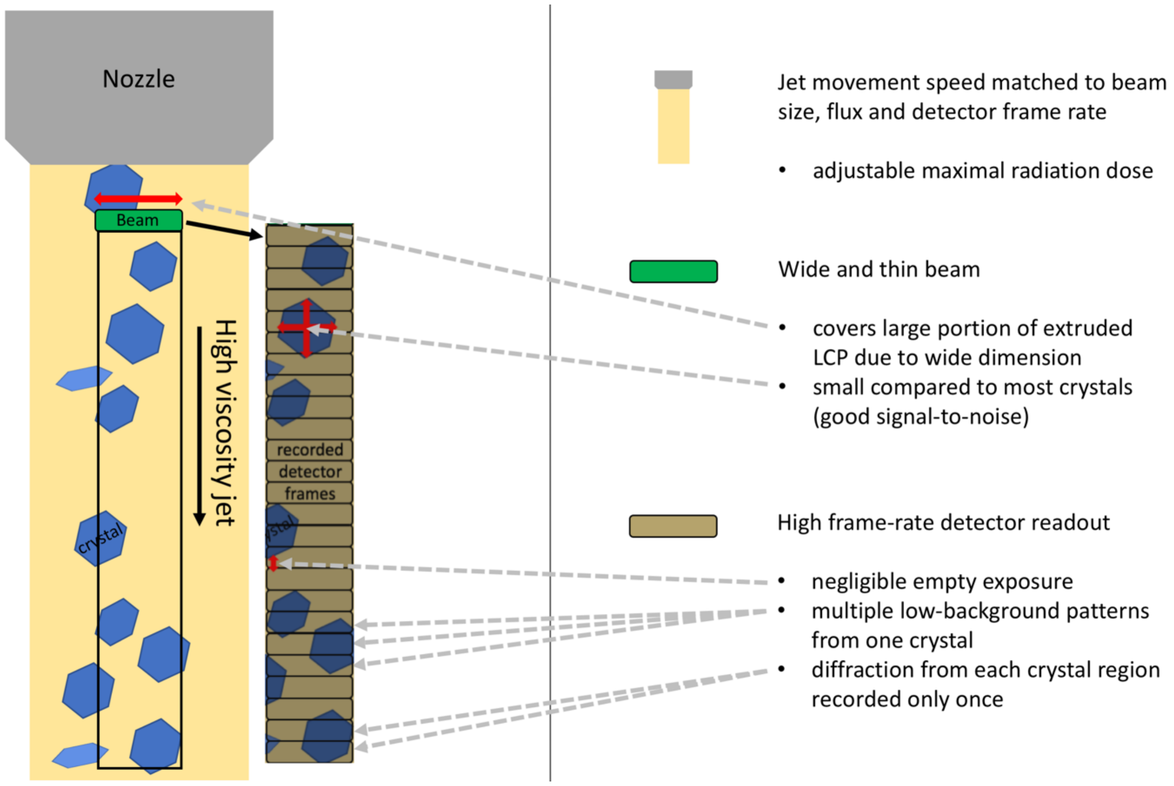

Supplementary Figure 4. Crystal scanning serial crystallography. The figure shows the advantages of the technique.

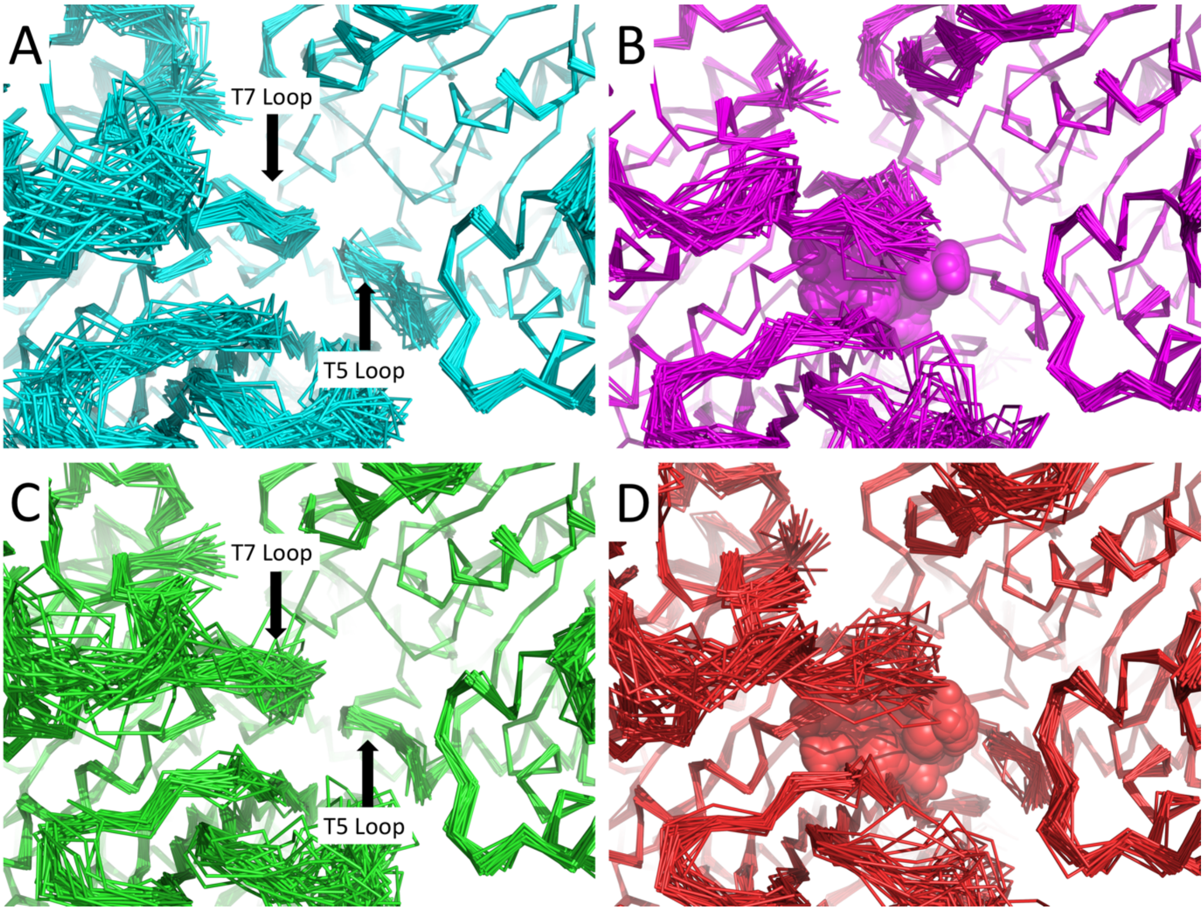

Supplementary Figure 5. Ensemble refinements of Tubulin structures at room temperature and at cryo- temperatures. (A) Ribbon representation of ensemble model of the cryo structure of TD1. (B) Ribbon representation of ensemble model of the cryo structure of TD1 with bound colchicine. (C) Ribbon representation of ensemble model of the room temperature structure of TD1. (D) Ribbon representation of ensemble model of the room temperature structure of TD1 with bound colchicine.

Supplementary Table 4. *Cryo Dataset of TD1$_{apo}$*

Dataset	TD1$_{apo}$ cryo
Beam size um	30 x 10
Flux (ph / s)	4 x 10^{11}
Crystal size µm^3	300 x 30 x 20
Oscillation range	0.1
Space group	$P2_1$
Unit cell (a, b, c in Å, β / γ in °)	74.0, 91.7, 82.6, 97.4
Indexed patterns	3600
Resolution	49.4 – 1.8
Number of Reflections	688123
Number of unique Reflections	100723 (7249)
Redundancy	6.8 (6.2)
Completeness	99.7 (99.2)
I / sigma	11.6 (0.7)
*CC**	0.99 (0.77)
CC1/2	0.996 (0.43)
Rmeas	10.8 (220.2)
R_{cryst} / R_{free}	17.6 / 20.7
Ramachandran favored / allowed / outliers	990 / 17 / 0
RMSZ (bonds)	0.49
RMSZ (angles)	0.63
Average B-factor	42.0
PDB Entry	5NQU

Supplementary Table 5. *cDNA and primer sequences*

type	**sequence**
DARPIN D1 cDNA	GACCTGGGTAAGAAGCTGCTGGAAGCCGCCCGTGCAGGTCAGGACGACGAGGTTCGTATCCTGATG<br>GCCAACGGCGCCGATGTTAACGCCACCGACGCCAGTGGTCTGACACCGTTACATCTGGCCGCCACC<br>TATGGTCACCTGGAGATCGTGGAGGTTTTATTAAAGCACGGCGCAGACGTGAACGCCATCGACATC<br>ATGGGCAGCACCCCGCTGCACTTAGCCGCCTTAATCGGTCACTTAGAAATCGTGGAAGTGCTGCTG<br>AAGCATGGCGCAGATGTGAACGCCGTTGACACCTGGGGTGACACCCCTCTGCATTTAGCCGCCATC<br>ATGGGCCACCTGGAGATTGTTGAGGTGTTACTGAAACACGGTGCCGACGTTAACGCCCAGGATAAG<br>TTCGGCAAGACCGCCTTCGACATCAGCATCGACAACGGCAACGAGGACCTGGCCGAGATCCTGCAG<br>AAGCTGAACTAA
Cloning Primer 1 for insertion into PSTCm8 vector	GTGCCGCGCGGATCCGACCTGGGTAAGAAGCTGCTGG
Cloning Primer 2 for insertion into PSTCm8 vector	TTGCTAAGTGAGCTCTGTCAATTATCAGTTCAGCTTCTGCAGGATCTCG
